# Inbreeding Calculated with Runs of Homozygosity Suggests Chromosome-Specific Inbreeding Depression Regions in Line 1 Hereford

**DOI:** 10.3390/ani11113105

**Published:** 2021-10-30

**Authors:** Bethany Pilon, Kelly Hinterneder, El Hamidi A. Hay, Breno Fragomeni

**Affiliations:** 1 Department of Animal Science, University of Connecticut, Storrs, CT 06269, USA; bethany.pilon@uconn.edu (B.P.); kelly.hinterneder@uconn.edu (K.H.); 2 College of Veterinary Medicine, Cornell University, Ithaca, NY 14853, USA; 3 College of Veterinary Medicine, Lincoln Memorial University, Harrogate, TN 37752, USA; 4 Fort Keogh Livestock and Range Research Laboratory, ARS, USDA, Miles City, MT 59301, USA; elhamidi.hay@usda.gov; 5 Institute for System Genomics, University of Connecticut, Storrs, CT 06269, USA

**Keywords:** inbreeding, runs of homozygosity, Hereford cattle

## Abstract

**Simple Summary:**

Inbreeding depression is the decreased fitness of offspring of closely related individuals. It is a common problem in the livestock industry as a consequence of intense selection favoring few animals with higher genetic merit. The literature shows that inbred individuals may also exhibit decreased performance for economically relevant traits, such as growth in beef cattle. However, this relationship is not simple. The use of genetic markers can improve the detection of homozygous regions and improve the calculation of inbreeding coefficients. The working hypothesis of this study is that genomic homozygous regions can be used to predict animals’ performance. Moreover, homozygous regions across the genome differ in their effects. Our results indicate that the relationship between homozygosity and decreased performance varies by chromosome. Additionally, the inbreeding coefficient can be used to predict animals’ phenotype when calculated within a chromosome. This research warrants the development of a new approach in genomic selection to increase prediction accuracy by including information on homozygous regions. Additionally, management tools can be developed with the goal of breeding individuals with a purpose to decrease homozygosity only in the relevant regions of the genome.

**Abstract:**

The goal of this study was to evaluate inbreeding in a closed beef cattle population and assess phenotype prediction accuracy using inbreeding information. Effects of inbreeding on average daily gain phenotype in the Line 1 Hereford cattle population were assessed in this study. Genomic data were used to calculate inbreeding based on runs of homozygosity (ROH), and pedigree information was used to calculate the probability of an allele being identical by descent. Prediction ability of phenotypes using inbreeding coefficients calculated based on pedigree information and runs of homozygosity over the whole genome was close to 0, even in the case of significant inbreeding coefficient effects. On the other hand, inbreeding calculated per individual chromosomes’ ROH yielded higher accuracies of prediction. Additionally, including only ROH from chromosomes with higher predicting ability further increased prediction accuracy. Phenotype prediction accuracy, inbreeding depression, and the effects of chromosome-specific ROHs varied widely across the genome. The results of this study suggest that inbreeding should be evaluated per individual regions of the genome. Moreover, mating schemes to avoid inbreeding depression should focus more on specific ROH with negative effects. Finally, using ROH as added information may increase prediction of the genetic merit of animals in a genomic selection program.

## 1. Introduction

Precision livestock farming relies on increased quality and quantity of data collected, leading to accurate decision making. With the rapid advancement in high-throughput technologies, the large amount of genomic information being generated can greatly contribute to this endeavor. In the beef cattle industry, with the use of genomic tools, we can dissect important genetic mechanisms and apply them to improve animal performance. A practical application of genomic information is its use in animal evaluation to increase phenotype prediction accuracy in younger animals [1], improve traits which are hard/expensive to measure, and traits with low heritability [2,3,4,5]. Increasing phenotype prediction accuracy leads to a higher genetic gain, which in turn improves animals’ efficiency and the overall sustainability of beef production systems. However, there are still many elements to be investigated regarding hybrid vigor, inbreeding, gene function and pathways, and how they relate to beef cattle production.

Line 1 Hereford is a unique and important population for the beef cattle industry. Many Hereford herds in the US and abroad have founders from this population [5]. Line 1 was founded using 2 half-sibling bulls and 50 unrelated cows and has remained a closed population since its formation [5,6]. From the inception of the line through 2010, genetic selection was limited to post-weaning growth. After 2010, no trait selection has been applied. Since the herd has been closed for over 80 years, the inbreeding coefficient (F) reached 0.30, which is considered high for beef cattle. An increase in 0.01 in the value of F can decrease weaning weight, yearling weight and average daily gain significantly [7]. In Angus, a similar pattern of inbreeding depression was found for growth traits [8], showing that inbreeding should warrant investigation in beef cattle populations.

Inbreeding coefficients calculated using runs of homozygosity (ROH) are the ideal approach to evaluate livestock [9]. ROH are long uninterrupted sequences of homozygous genotypes over regions of the chromosome and provide reliable estimates of F values [10,11] (Additionally, Howard et al. [12] suggested a novel method to identify inbred genomic regions associated with reduced performance in swine. Finally, Curik et al. [13] has shown that ROH frequencies vary widely within and across chromosomes.

Taking together the information from the literature, the working hypothesis for this study is that animals are affected differently by inbreeding, which suggests that homozygosity might result in different effects across individuals. The goal of this study is to evaluate the effect of inbreeding and phenotype prediction accuracy using chromosome-specific ROHs in the Line 1 Hereford population.

## 2. Materials and Methods

Average daily gain (ADG) (h^2^ = 0.25) was analyzed based on the results of Sumreddee et al. [7]. The reason for choosing this trait is because it was the phenotype with larger effects of inbreeding depression and higher data availability. The data were filtered to remove outliers (±3 standard deviations) to ensure that the analyzed phenotypic values were relevant. Additionally, phenotypes with 0 value were also excluded, as they were considered to be missing data. A total of 672 animals born between 1953 and 2016, genotyped with a range of low to medium SNP density panels (3 K to 50 K SNPs) were used in this study. Quality control of genotype data consisted of filtering out SNPs with a call rate smaller than 90%, a minor allele frequency (MAF) less than 5%, a heterozygous deviation greater than 15% from the Hardy–Weinberg Equilibrium. Call rates below 90% for animals were investigated; however, all individuals passed the test. Animals genotyped with low-density chips (i.e., 3 K, 9 K, 20 K, and 27 K) were imputed to the 50 K marker panel using pedigree-based parameters, implemented in the FImpute software [14]. Imputation details and accuracy in addition to the population structure of line 1 Hereford were investigated by Huang et al. [15] and Huang et al. [16]. After quality control and imputation, the total number of genotyped animals remained at 672 and SNPs were reduced to 30,810.

The mean and standard deviation for ADG were 0.83 kg/day and 0.353 kg/day, respectively. Phenotypes were corrected by the fixed effects of year of birth and sex, and all models below used the corrected phenotype as the dependent variable, unless raw phenotypes were specified.

Inbreeding coefficients (FPED) were estimated based on the probability of identity by descent that occurred at random loci, calculated with pedigree-based analysis. The tabular method (Henderson, 1976) was used to calculate FPED as implemented in the pedigree R package version 1.4 [17] using R [18] version 3.12. Additionally, as autozygosity across chromosomal segments can be measured based on ROHs, genomic information was used to calculate ROH-based inbreeding coefficients (FROH) using SNP data. The DetectRUNS R package version 0.9.6 was used to identify ROH segments based on SNP data [19]. To identify runs of homozygosity, short and common ROH segments less than one Mb in length, which occurred frequently throughout the genome, were excluded. The minimum ROH length used was 30 SNPs and the minimum density used to detect ROHs was 1 SNP in every 500 kb. In an ROH, a maximum of two heterozygous and two missing SNPs were allowed. The sliding window-based method was used to define FROH for each genotyped animal. To determine the effect of chromosome-specific inbreeding, FROH of an individual was further partitioned into the relative contribution of each ith chromosome (FROH-CHRi), computed as a ratio between the length of the chromosome covered by ROHs and the total length of the chromosome. The data were filtered to include only FROH values greater than 0.

To estimate inbreeding depression and the effect of inbreeding coefficients in the phenotypic studies, the following model was fitted:

$$y_{i}=\mu +f_{i}*a+e_{i}$$

where $y_{i}$ is the phenotype for animal *i*, *μ* is the intercept, $f_{i}$ is the inbreeding coefficient for animal *i* calculated based on pedigree information (FPED), ROH (FROH), or chromosome-specific data (FROH-CHRj, where j represents the jth chromosome), a is the regression coefficient of $y_{i}$ on $f_{i}$ (FPED, FROH, or FROH-CHRj), and $e_{i}$ is the residual associated with each observation.

Inbreeding depression was estimated by regressing trait phenotypes on the ROH-inbreeding coefficient. The prediction ability of each model was assessed as the correlation between the predicted phenotype by the linear model above and the observed phenotype of the animals in the population. The term accuracy was used to describe this prediction ability, while the slope of the regression coefficient for each chromosome was used to estimate the effect of inbreeding on the phenotype. The chromosome-specific inbreeding depression was evaluated as the average slope of the FROH-CHRi for each chromosome.

To calculate the prediction accuracy, data were randomly split in two groups, where 80% of the observations were termed training group and the remaining 20% were referred to as the validation group. On average, 538 and 134 individuals were in the training and validation group, respectively. The regression was fit using the training data and the regression coefficients were then used to predict the regression validation values which represent animals with masked phenotypes. The correlation between the predicted values and the observed values on validation animals were then calculated and used to validate the model and demonstrate the ability in predicting phenotypic values, corrected for the systematic effects of year and sex. Using inbreeding coefficients calculated with pedigree and ROH coverage across the entire genome or chromosome-specific ROH, the process was repeated 500 times to decrease potential sampling error and identify highly variable predictors. Mean and standard deviation of accuracy across the 500 samples were used to assess prediction ability. The reason for using 500 samples was the stability of the model. Higher fluctuations on values were observed when fewer samples were included, while convergence was always achieved with 500 rounds. Convergence was assessed as changes smaller than e-04 on the average accuracy.

The contribution of each chromosome to the phenotype was compared using each FROH-CHRi in a separate model. Another model was fit including all 30 chromosomes as separate variables. For the X chromosome, males ROH were considered missing data, and only females were used in the accuracy computing. Finally, only the chromosomes with higher accuracy, lowest *p*-value or highest effect were fit in another set of models. For the models with selected chromosomes, the regression coefficient and the *p*-value used to select chromosomes were calculated using the complete data.

The difference between the mean accuracy of each group was calculated and the Tukey honest significance test [20] was used to assess pairwise differences between sample means. *p*-values less than 0.05 indicate a significant difference between the pair of models.

## 3. Results and Discussion

The average pedigree inbreeding was 0.29, and the average ROH inbreeding was 0.32. The correlation between the inbreeding coefficients was 0.6. These values were similar from those obtained by Sumreddee et al. [7], and the differences were due to the parameters for calculating ROH inbreeding. Because the effects of year of birth and sex were also significant in an initial analysis, only inbreeding depression results with corrected phenotypes are shown. Both FPED and FROH effects were significant (*p* < 0.01) on ADG, and inbreeding depression was stronger from FPED than from FROH, −1.3 and −0.9 kg/day, respectively. These values cannot be directly compared with those by Sumreddee et al. [7], because of the use of corrected phenotypes in the present study.

The prediction accuracy for the raw phenotypes reached higher values when sex and year of birth were included in the model. However, the prediction accuracy of pedigree (FPED) and genomic (FROH) inbreeding coefficient approached 0 when corrected phenotypes were the dependent variable. Moreover, the gains in accuracy from inbreeding coefficients on raw phenotypes were marginal when compared to the effects of sex and year of birth, even when significant (Table 1). Those results showed a strong confounding between systematic effects and prediction accuracy. The effects from year of birth were expected, given the increasing inbreeding coefficients with time, as shown by Sumreddee et al. [7]. Sex effects were also expected to affect average daily gain, given well-known differences between males and females growth.

**Figure 1:**
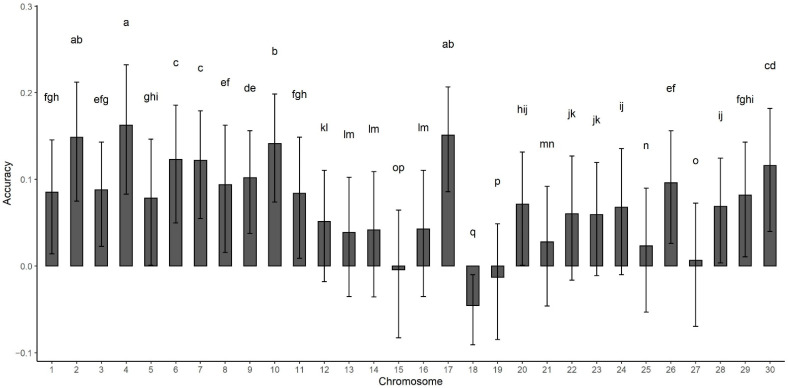
Prediction accuracy of run of homozygosity calculated within each individual chromosome. Letters a-n represent pairwise comparisons among chromosomes. Chromosomes not sharing any letter in the notation above the error bar are significantly different by Tukey test (*p* > 0.05). Error bars represent the standard deviation across 500 cross validation rounds. Chromosome X is hereby termed 30.

From these analyses, the lack of prediction power by inbreeding coefficient was clear. On the other hand, the impact of specific chromosomes on phenotype prediction varied across the 30 bovine chromosomes, as shown in Figure 1. Different from ROH calculated using the entire genome, individual chromosomes presented positive prediction power. Depending on the chromosome, the accuracy varied from approximately 0 to 0.2 (Figure 1). Previous studies identified genomic regions in ROH associated with reduced performance in dairy cattle [21,22]. However, they did not calculate the prediction power of these regions. The authors found inbred regions that were under positive selection, and were associated with desired traits, showing that favorable alleles may become fixed in a population [22]. Additionally, Howard et al. [12] developed an algorithm that identifies unfavorable haplotypes within a ROH and presented prediction accuracy greater than 0. In the present studies only chromosomes FROH were fit, which differs from Howard et al. [12]. However, both findings conclude that weighting inbred regions differently offers potential to perform genomic prediction.

Comparison of the similarities between the 30 genotyped chromosomes indicated that the phenotype can be predicted based on the unique contributions of each individual chromosome. Chromosomes 15, 18, 19, and 27 were found to have very low accuracies while the highest accuracies were found on chromosomes 2, 4, 10, and 16. Chromosome X demonstrated intermediate to high accuracy; however, this should be carefully interpreted as only females had meaningful ROH values for this chromosome. There is no evident relationship between the accuracy obtained by individual chromosomes and the number or coverage of QTL in the Cattle QTL Database [23].

The prediction power fitting all 30 chromosomes individually was higher than calculating ROH for the whole genome. Selecting the chromosomes with higher and significant effects increased prediction accuracy even further (Table 2). Adding chromosomes with smaller accuracy did not achieve the maximum prediction power, which shows that this approach is sensitive to multicollinearity.

**Figure 2:**
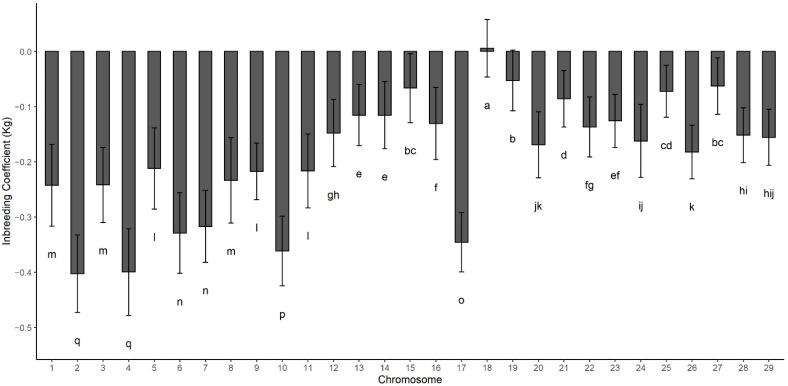
Inbreeding depression calculated in each of the 29 bovine autosomes. Letters a-n represent pairwase comparisons among chromosomes. Chromosomes not sharing any letter in the notation below the error bar are significantly different by Tukey test (*p* > 0.05). Error bars represent the standard deviation across 500 cross validation rounds.

The inbreeding depression values for individual chromosomes is presented in Figure 2 and ranged from close to 0 in chromosome 18 to approximately −0.2 Kg/day on chromosome 2, 4, 10, and 17. Inbreeding depression for chromosome X resulted in estimates outside the expected parametric space (results not shown) and were excluded. There is a connection between accuracy and inbreeding depression, but it does not seem to show a perfect relationship. There is a weak association between chromosome length and inbreeding depression. Nonetheless, the relationship is not strong, and there are outliers, such as chromosome 1, 3, and 17. Such findings emphasize the differences in how inbreeding affects phenotypes and how important it is to account for specific regions when studying runs of homozygosity and inbreeding [24].

Associations between chromosomal regions and growth traits in beef cattle were identified by several authors [25,26,27,28,29]. The current findings are consistent with the literature, suggesting a non-uniform distribution and effect of contributing variants across the bovine genome, even for complex traits. Additionally, the variation on accuracy for each chromosome agrees with Maltecca et al. [30] that not all inbreeding is deleterious. Using the whole genome ROH coverage to predict inbreeding depression may be misleading and result in biased estimates, as some ROH can have positive impacts on phenotypes [12]. In a population with high inbreeding levels for a long time, such as Line 1 Hereford, the effects of inbreeding depression could be moderate because of selection against deleterious alleles in the population [31]. As an illustration of that in a bubaline population, Macciota et al. [32] used ROH islands to identify genes associated with environmental adaptation, fitness, and reproduction. In this sense, positive and negative inbreeding may counterbalance, leading to small or no inbreeding depression, as found by Sumreddee [7] for reproduction traits.

Studies with ROH in dairy cattle have shown that chromosomal ROH vary widely [21,22,23,24,25,26,27,28,29,30,31,32,33]. Similarly, in beef cattle the contributions of different chromosomes also fluctuate [7,34,35]. The variation in effect and distribution of deleterious ROH suggests that a mating design can be devised to reduce inbreeding depression, similarly to what is suggested by VanRaden et al. [2] with lethal haplotypes. This breeding scheme may be adopted per chromosome ROH, and can be further developed for specific ROH to be avoided. Due to the complexity of several traits of interest and the selection applied to animals, it is likely that there is a large number of ROH regions with small effects. As discussed in Howard et al. [22], most regions identified in dairy cattle had a small effect, resembling a polygenic trait GWAS. Therefore, using the most common ROHs in the population as selected variants in the SNP panel may be a practical alternative as it has been demonstrated in single-step GBLUP [36,37] and Bayesian hierarchical models [38,39].

The results obtained in this study conflict with the findings by Doekes et al. [40] in Dutch Holstein–Friesian dairy cattle. The authors found that inbreeding depression was distributed uniformly across the genome, and that inbreeding-specific regions would provide little benefit to genomic prediction methods. The most likely reason for the differences obtained is the population structure for Line 1 Hereford and the higher inbreeding coefficients. Cesarani et al. [41] identified 34 genomic regions associated with milk production traits in an European population. While the results from this study resembles a mostly polygenic scenario, it illustrates that distribution and effects of ROH are data and population dependent. Similarly, Martikainen et al. [42] were able to identify associations between ROH and some genomic regions in Finnish Ayrshire cattle.

An additional aspect that can affect findings is the parameters to define a homozygous region as an ROH. Shorter segments are related to ancient inbreeding and longer segments to recent inbreeding [43], and the choice of parameters in the software should prioritize a minimum length of the ROH. This could lead to many regions with false positives, when aiming to identify ancient inbreeding, or ignore some shorter runs, when the objective is to find recent inbreeding. Additionally, for shorter ROHs, SNP data may introduce biases and not identify some regions [9]. On the other hand, ancient inbreeding is less likely to cause deleterious effects because of selection against deleterious alleles, which indicates that future research should focus on medium to long ROHs. Novel methods to distinguish between new and old inbreeding are being developed [7] and should be used in future applications.

## 4. Conclusions

Inbreeding coefficients calculated with the entire genome had no prediction ability, despite the significance of the regression model. When fitting ROH calculated in individual chromosomes, they were found to differ in accuracy and contribute differently to inbreeding depression. Results from the present study show evidence that not all homozygous regions affect phenotypes equally, and that should be accounted for when studying inbreeding depression. Moreover, these results suggest that future studies should test individual ROH as prediction coefficients, as they may contribute to increased accuracy in genomic evaluation models. While the prediction accuracy using only ROHs was not high, it suggests that individual-specific inbred chromosomal regions may be used to increase the prediction ability of genomic selection models. Finally, different chromosomes were found to have different inbreeding depression levels, suggesting that the severity of inbreeding effects varies across the genome.

## Figures and Tables

**Table 1:** The mean prediction accuracy and standard deviation of each model using raw (ADG) or correct (ADGC) phenotypes.

Model *	**Accuracy**	**SD**
ADG = F_PED_	0.17 ^e^	0.05
ADG = F_PED_ + Sex	0.76 ^c^	0.02
ADG = F_PED_ + Sex + YOB	0.88 ^a^	0.02
ADG = F_ROH_	0.21 ^d^	0.05
ADG = F_ROH_ + Sex	0.77 ^b^	0.02
ADG = F_ROH_ + Sex + YOB	0.88 ^a^	0.02
ADG = Sex	0.76 ^c^	0.03
ADG = Sex + YOB	0.88 ^a^	0.02
ADG_C_ = F_PED_	0.01 ^f^	0.3
ADG_C_ = F_ROH_	0.02 ^g^	0.4

* The independent variables sex, year of birth (YOB), pedigree inbreeding coefficient (FROH), and runs of homozygosity inbreeding coefficient (FROH) were considered. Different letters represent that means differ significantly (*p* > 0.05).

**Table 2:** Accuracy mean and standard deviation (SD) from inbreeding coefficients using selected chromosomes based on significance of the effects.

Model	Accuracy	SD
ADG_C_~CHR1-CHRX	0.13	0.06
ADG_C_~Chr1 + Chr4 + Chr6 + Chr7 + Chr10 + Chr15 + Chr17 + Chr22	0.19	0.06
ADG_C_~Chr1 + Chr4 + Chr6 + Chr7 + Chr10 + Chr15 + Chr17 + Chr22 + ChrX	0.19	0.06
ADG_C_~Chr4 + Chr7 + Chr10 + Chr15 + Chr17 + Chr18 + ChrX	0.22	0.06
ADG_C_~Chr7 + Chr10 + Chr17 + Chr18 + ChrX	0.21	0.06

## Data Availability

The data supporting the findings of this study are available upon request from the author El Hamidi Hay: elhamidi.hay@usda.gov and with permission from the USDA Agricultural Research Service.
